# Non-Steroidal Anti-Inflammatory Drugs in Orthopedic Outpatient Cases in a Tertiary Care Teaching Hospital: A Descriptive Cross-sectional Study

**DOI:** 10.31729/jnma.6767

**Published:** 2021-11-30

**Authors:** Ashish Kumar Bhattarai, Krishna Raj Khanal, Jyoti Prabha Bharati

**Affiliations:** 1 Department of Pharmacology, Kathmandu Medical College, Duwakot, Bhaktapur, Nepal; 2 Department of Orthopedics, Kathmandu Medical College, Sinamangal, Kathmandu, Nepal

**Keywords:** *anti-inflammatory drugs*, *drug utilization*, *non-steroidal*, *proton pump inhibitor*, *rabeprazole*

## Abstract

**Introduction:**

Non-steroidal anti-inflammatory drugs are major drugs in treatment of pain and inflammation of different orthopedic conditions. There are different classes of non-steroidal antiinflammatory Drugs based on their selectivity to cyclooxygenase enzyme which has significant differences in safety profile. This study aims to determine the prevalence of non-steroidal antiinflammatory drugs prescription in the orthopaedic outpatient department of a tertiary care hospital.

**Methods:**

This was a descriptive cross-sectional study conducted among the patients in the orthopedic outpatient department of tertiary care hospital from December 2020 to March 2021. Ethical approval was taken from the Institutional Review Committee of the college (Ref: 0311202007). Convenient sampling was done. A structured proforma was used with consent. The data were analyzed with Social Statistical Package for the Social Sciences version 20. Point estimate at 95% Confidence Interval was done and frequency and percentage were calculated.

**Results:**

Out of 140 orthopaedic out patient department prescriptions screened, 118 (84.28%) (78.2590.30 at 95% Confidence Interval) prescriptions included non-steroidal anti-inflammatory drugs. Aceclofenac 76 (64.4%) was the most prescribed non-steroidal anti-inflammatory drug.

**Conclusions:**

Aceclofenac was the most preferred agent for the treatment in the department of orthopedics. The most common proton pump inhibitor used alone with non-steroidal antiinflammatory drugs was rabeprazole.

## INTRODUCTION

The treatment of pain and inflammation is an important aspect in orthopedic cases. Non Steroidal AntiInflammatory Drugs (NSAIDs) are the major drugs in the treatment of pain and inflammation.^1^

NSAIDs exert their analgesic and anti-inflammatory action by inhibiting the enzyme cyclooxygenase (COX), which are responsible for the generation of different types of prostaglandin analogs. The anti-inflammatory effects of the NSAIDs are by inhibition of inductive COX-2, while different side effects are attributed to inhibition of Constitutive COX-1 isoform.^1^ Due to the availability of safer NSAIDs like selective COX-2 inhibitors and difference in efficacy and tolerability the prescription pattern is frequently changed.^2^

This study aimed at determining the prevalence of non-steroidal anti-inflammatory drugs prescription in the Orthopaedic Out patient department of a tertiary care hospital.

## METHODS

This was a descriptive cross-sectional study conducted in the Kathmandu Medical College Teaching Hospital. It was conducted among the patients visiting at the orthopedic department of this hospital from December 2020 to March 2021. This study was approved by the Institutional Review Committee of the Kathmandu Medical College (Ref: 0311202007). The patients coming to orthopedic treatment with age group above 18, willing to participate in the study were included. Those patients not willing to participate and the prescriptions not containing NSAIDs were excluded from the study. Convenient sampling was done and the sample size was calculated using the formula,

n = Z^2^ × p × q / e^2^

  = 1.96^2^ × (0.5) × (1-0.5) / (0.1)^2^

  = 97

where,

n= sample size
Z= 1.96 at 95% Confidence Interval
p= prevalence taken as 50%
q= 1-p
e= margin of error, 10%

The calculated sample size was 97. Taking the nonresponse rate of 10%, the sample size was 107. However, we included 140 participants.

After getting permission from the IRC and department of Orthopedic, the study was conducted in the orthopedic department. All the prescriptions comprising NSAIDs were recorded. The informed written consent was taken from the entire participants. This was done till desired sample size was achieved. Prescriptions containing NSAIDs were documented in the specialized proforma (Annexure I and II). The proforma was finalized by the faculty members of the department of Pharmacology and department of orthopedics for content validation. The proforma was pretested among a small number of ten participants for the understanding and acceptance for the face validation. These participants were not included for the study. Based on the response of the pretesting, after minor modification, the proforma was finalized and distributed.

The proforma Annexure I consisted of demographic profile, diagnosis and medical history of the patient. Annexure II constituted, the name of NSAIDs, number of drugs prescribed, number of drugs in Essential Drug list, Adjuvant and corrective drug given.

Data was collected, compiled and analyzed by using the Statistical Package of social science (SPSS) version 20. Point estimate at 95% Confidence Interval was done and descriptive statistics were calculated.

## RESULTS

The total number of prescriptions included in the study was 140, and among these 118 (84.28%) (78.20-90.36 at 95% Confidence Interval) prescriptions constituted NSAIDs. The total number of drugs prescribed in the prescription containing NSAIDs was 264. The number of fixed drug combinations (FDC) where NSAIDs was one of the constituents was 6 (4.28%). Mean number of drugs per prescription was 2.32±0.8.

Of the 118 total participants, male subjects 60 (50.85%) were slightly more than female subjects 58 (49.15%). The mean age of the subjects was 45.5±16.16 (Table 1).

**Table 1:** Gender distribution among NSAID prescribed orthopaedic OPD prescriptions.

Gender	n (%)
Male	60 (50.85)
Female	58 (49.15)

Among the 118 prescriptions with NSAIDs, 76 (64.40%) aceclofenac was the most prescribed drug. The details of NSAIDs prescription (Table 2).

**Table 2:** Details of NSAIDs prescription.

Name of drugs	n (%)
Aceclofenac	76 (64.40)
Naproxen	16 (13.56)
Ibuprofen	10 (8.47)
Indomethacin	7 (5.93)
Combinations (FDCs)	6 (5.08)
Diclofenac	3 (2.54)
Total	118 (100)

The drugs which were given to correct the possible side effects of the NSAIDs were referred to as corrective drugs. A total of 102 (86.44%) of the total patients were prescribed the corrective drug. Most of the patients were prescribed rabeprazole 90 (88.23%) while pantoprazole and ondansetron were prescribed to 6 (5.88%) patients (Table 3).

**Table 3:** Corrective drug usage.

Name of drug	n (%)
Rabeprazole	90 (88.23)
Pantoprazole	6 (5.88)
Ondansetron	6 (5.88)
Total	102 (100)

## DISCUSSION

NSAIDs are among the oldest drugs. Some of the NSAIDs are safe enough to be sold over the counter while many NSAIDs require a prescription. NSAIDs are associated with many adverse reactions, morbidity and even death of the patients. For the physician, consideration of safety, cost and availability is important before prescribing NSAIDs. Most adverse reactions associated with NSAIDs are dose dependent. The newer NSAIDs has advantage of more safety retaining the efficacy as traditional NSAIDs.^1^ NSAIDs inhibit enzymes responsible for synthesis of different prostanoids prostaglandins, prostacyclin, and thromboxane and cyclooxygenase (COX), existing in 2 isoforms (COX-1 and COX-2). COX-1 is constitutively expressed by most cells, leading to the production of prostanoids (like prostacyclin PGI2) that play a housekeeping role in the maintenance of normal renal function, platelet aggregation, and gastric mucosal integrity. COX-2 can be expressed both constitutively and in response to inflammatory stimuli and is responsible for the generation of prostanoids important for inflammation.^4^ Based on their difference in potency to inhibit COX-1 and COX-2, NSAIDs are defined as non selective NSAIDs which predominantly inhibit COX-1 and to a lesser extent COX-2. Newly developed compounds that predominantly inhibit COX-2 or selective COX-2 inhibitors strong inhibitors of inflammatory prostanoids, but only slightly affect the production of protective prostanoids generated.^5^ The study of drug utilization patterns for NSAIDs can be helpful for rational and evidence based prescribing tools to choose the right drug dosage for any medical condition.

In this study 90% of the prescription volume mainly consisted of 4 drugs namely Acelofenac, Naproxen, Ibuprofen and Indomethacin. DU90% is an innovative way to assess drug prescribing. Using this approach the drugs that represent 90% of the drug prescription/ sales volume are identified. Less the number of drugs in the DU90% segment, more rationale is the prescribing pattern. Furthermore, the approach can be used to assess what proportion of the drugs that represent 90% of the volume is made up of drugs listed in the essential drug list.^3^ DU 90% provides pertinent information on drug usage in patients and could be widely applied as a basis for preparing prescription guidelines. Contrary to this result selective COX-2 inhibitor Celecoxib was mainly prescribed. Arthritis was the most common cause of taking NSAIDs (67%) in this study followed by spinal disease (9%), fracture (5%), sprain (4%), other inflammatory diseases (4%), and others (12%).^6^

NSAIDs utilization study conducted in Queens land and all of Australia shows that before the introduction Of COX-2 selective inhibitors, non-selective-NSAIDs were the major component of DU90% (1997-99).^7^ Similarly in PGI Chandigarh, before the withdrawal of rofecoxib, COX-2 inhibitors were the major component DU90%.^8^ When COX-2 selective inhibitors took over, these non-selective NSAIDs were progressively replaced and celecoxib and rofecoxib became the major component of DU90% (2000-03).^9^ The withdrawal of rofecoxib by the manufacturing company, due to major cardiovascular side effects, is likely to change the prescribing pattern of NSAIDs.

The prescription pattern of NSAID saw frequent changes under the influence of new study revelation on safety data, clinical trial results and frequent changes in the guidelines of NSAID prescription. Starting from the use of traditional NSAIDs in the beginning; to more selective COX-2 inhibitors later and then again reversing back to traditional NSAIDs after withdrawal of rofecoxib. Recent research questioning the cardiovascular safety profile of traditional NSAIDs has added further confusion with regard to rational NSAID prescription.^9^ After the withdrawal of COX-2 inhibitors rofecoxib and valdecoxib, there were significant increases in non-selective NSAID and PPI prescriptions.^10^

According to a summary of recommendations from The Third Canadian Consensus Conference (2005) regarding an evidence-based approach to prescribing NSAIDs, If NSAIDs must be used in high-risk patients with a history of gastrointestinal bleeding; a proton pump inhibitor should be prescribed as well.^11^ About 6.44% of the patients were prescribed the corrective drug to prevent the gastrointestinal side effects of the NSAIDs. Most of the patients were prescribed Rabeprazole 88.23% while Pantoprazole and ondansetron were each prescribed to 5.88% of patients as shown in Table 3. Ondensatrone was prescribed to the patients who were given tramadol as analgesic.

According to one study, 38% of Gastrointestinal (GI) bleeding is associated with NSAIDs. But the risk of gastrointestinal complication with Acelofenac was very low (1.4%).^12^ The drug accounting maximum prescription in our study was Acelofenac (64.4%). Although the G.I side effect with Acelofenac is minimal, about 86.4% were given Proton pump inhibitors (PPI). Hence, further extensive study is suggested for finding association of PPI and NSAIDs rational prescription. The limitation of the study is that it was conducted in a small population and one hospital only, so the findings from this study cannot be generalized for the whole population.

## CONCLUSIONS

This descriptive study shows that the most prescribed drug in the orthopedic outpatient department is Acelofenac. Most prescribed drugs were Acelofenac, Naproxen, Ibuprofen and Indomethacin. The usage of fixed dose combinations was relatively low. In most cases the prescription proton pump inhibitor was prescribed alone with the NSAIDs. Rabeprazole was most preferred among proton pump inhibitors. For more conclusive and convincing finding a multi centric study with sufficient sample size has to be taken. And the study modalities should be elaborated extensively.
